# Environmental Issues in Thyroid Diseases

**DOI:** 10.3389/fendo.2017.00050

**Published:** 2017-03-20

**Authors:** Silvia Martina Ferrari, Poupak Fallahi, Alessandro Antonelli, Salvatore Benvenga

**Affiliations:** ^1^Department of Clinical and Experimental Medicine, University of Pisa, Pisa, Italy; ^2^Department of Clinical and Experimental Medicine, University of Messina School of Medicine, Messina, Italy; ^3^Master Program of Childhood, Adolescence and Women’s Endocrine Health, University of Messina School of Medicine, Messina, Italy; ^4^Interdepartmental Program of Molecular & Clinical Endocrinology, and Women’s Endocrine Health, University Hospital, Policlinico G. Martino, Messina, Italy

**Keywords:** autoimmune thyroid diseases, genetic influences, environmental influences, thyroid cancer, antithyroid antibodies

## Abstract

Environmental factors are determinant for the appearance of autoimmune thyroid diseases (AITD) in susceptible subjects. Increased iodine intake, selenium, and vitamin D deficiency, exposure to radiation, from nuclear fallout or due to medical radiation, are environmental factors increasing AITD. Cigarette smoking is associated with Graves’ disease and Graves’ ophthalmopathy, while it decreases the risk of hypothyroidism and thyroid autoimmunity. Viral infections are important environmental factors in the pathogenesis of AITD, too, particularly human parvovirus B19 (EVB19) and hepatitis C virus. Among the many chemical contaminants, halogenated organochlorines and pesticides variably disrupt thyroid function. Polychlorinated biphenyls and their metabolites and polybrominated diethyl ethers bind to thyroid transport proteins, such as transthyretin, displace thyroxine, and disrupt thyroid function. Among drugs, interferon- and iodine-containing drugs have been associated with AITD. Moreover intestinal dysbiosis causes autoimmune thyroiditis. To reduce the risk to populations and also in each patient, it is necessary to comprehend the association between environmental agents and thyroid dysfunction.

## Introduction

Graves’ disease (GD) and Hashimoto’s thyroiditis (HT) are the principal clinical presentations of autoimmune thyroid diseases (AITD), characterized by lymphocytic infiltration of the thyroid parenchyma, and clinically by thyrotoxicosis and hypothyroidism, respectively. AITD lead to an immune attack on the thyroid, whose mechanisms are still unclear. Genetic susceptibility and environmental triggers interact as the principal active part toward the development of the disease.

Autoimmune thyroid diseases prevalence is about 5% (1, 2) while that of antithyroid antibodies (ATAs) without clinical disease could be higher (3).

## Endogenous Factors Associated with AITD

Autoimmune thyroid diseases commonly affect more frequently females than males, such as in many other autoimmune diseases (4).

This is probably due to differences between male and female immune systems (5), which are present in many animal species; in fact, males have immune suppression versus females, which is linked to male sexual activity (6). Females show greater immune reactivity (7), and this increased immunocompetence might translate to greater resilience to infectious and non-infectious disorders.

In females, the immunological changes during pregnancy and their regression in the postpartum period are determinant, even if the predisposition of females to AITD is observed in nulliparous women, too. Microchimerism (i.e., the presence of small populations of cells from one subject in another genetically distinct one) is an endogenous factor associated with AITD (8).

Antithyroid antibodies frequency has a peak around 45–55 years, and hypothyroidism due to HT is more frequent in advanced age.

The HT prevalence and that of ATAs differ among races (8–10).

Incidence of HT shows an elevated geographic variability. However, worldwide, the current incidence rates of hypothyroidism and HT are higher than previously in similar regions (11); it is unclear whether this depends on the use of more accurate diagnostic procedures or actual increased incidence *per se* (12).

## Genetic Susceptibility

The importance of genetic susceptibility has been suggested by the familial clustering of AITD (20–30% in siblings of affected subjects, with a sibling risk ratio of approximately 17; high prevalence of ATA (about 50%) in siblings of AITD patients) (13). The concordance rate of monozygotic twin studies is ranging from 0.3 to 0.6 versus 0.00–0.1 for dizygotic twins (8, 14); heritability of ATA is 70%, of GD 79% (8).

Among genes linked to AITD and/or the presence of ATA (15, 16), whose function is known, 7 out of 11 take part in T cells function, leading to hypothesize that T lymphocytes are important in the pathogenesis of AITD.

## Environmental Factors

Environmental factors influence the occurrence of AITD of approximately 20%, as they are associated with the activation of innate immune response and AITD development in susceptible individuals (17, 18).

### External Radiation

The prevalence of ATA increased in children and adolescents exposed to radiations about 6–8 years after the Chernobyl accident. Thyroid peroxidase antibodies (TPOAb) prevalence was higher in radiation-exposed Belarusian children (6.4 versus 2.4% in not exposed) and in adolescents exposed to radioactive fallout 13–15 years after the Chernobyl accident (19). The radiation dose was related to increased TPOAb prevalence, thereby suggesting that important clinical changes could appear gradually (20). The onset of GD, and even of Graves’ ophthalmopathy (GO), may occur after radioiodine treatment of toxic goiter (21). Thyroid ionizing radiation exposure was related with the occurrence of tardive hypothyroidism, thyroid nodules, or cancer, and even acute thyroiditis (22, 23).

### Iodine

Iodine is essential for thyroid function. Constant iodine prophylaxis and increased iodine intake gradually reduce iodine deficiency-related thyroid disorders (24). In the Danish population, a 53% higher incidence of spontaneous overt hypothyroidism (probably autoimmune) was observed in mild, than in moderate, iodine deficiency (25). Iodine excess, owing to a great environmental iodine exposure besides poor monitoring, is a precipitating environmental factor in the development of AITD. Excessive amounts of iodide have been associated with the onset of autoimmune thyroiditis (AT) (26).

### Selenium

Selenium, whose necessary intake ranges from 60 to 75 μg/day, exerts an influence on immunological responses, cell growth, and viral defense and is necessary for normal thyroid function and homeostasis in humans. Selenium is essential for the activity of enzymes, such as glutathione peroxidases, deiodinases, and thioredoxin reductases, and for the synthesis and function of thyroid hormones and protects cells against free radicals and oxidative damage. A low selenium concentration is associated with the appearance of AT or GD (27). As selenium is involved in regulation of cell cycle, a decreased concentration is also important in the development of thyroid cancer (TC) (28, 29). In a cross-sectional observational study in Shaanxi Province (China), the relationship between selenium status, dietary factors, and pathological thyroid conditions was investigated. In the adequate-selenium county, the prevalence of pathological thyroid disorders (subclinical hypothyroidism, hypothyroidism, AT, and enlarged thyroid) was significantly lower than in the low-selenium county (18.0 versus 30.5%; *P* < 0.001). Elevated circulating selenium level was associated with reduced odds ratio (95% confidence interval) of subclinical hypothyroidism (0.68; 0.58, 0.93), hypothyroidism (0.75; 0.63, 0.90), AT (0.47; 0.35, 0.65), and enlarged thyroid (0.75; 0.59, 0.97) (30).

A paper (31) evaluated the real efficacy of selenium supplementation in HT, measuring thyroid-stimulating hormone (TSH), thyroid hormones, TPOAb and thyroglobulin antibodies (TgAb) levels, and thyroid echogenicity after 6 months of l-selenomethionine treatment. The authors concluded that the short-term l-selenomethionine supplementation has a restricted impact on the natural course in euthyroid HT.

### Smoking

The data about the effect of smoking on ATA (TgAb and TPOAb) and chronic AT (32, 33) are conflicting.

A meta-analysis reported the association of smoking with HT and postpartum thyroid dysfunction (34). By contrast, a prospective cohort study demonstrated, in subjects at risk of developing AITD, that smoking was negatively associated with the presence of TPOAb (35, 36), which was confirmed in population studies (37–40). Smoking during pregnancy was inversely correlated with the risk of thyroiditis, while it raised the prevalence of postpartum thyroiditis (41).

On the whole, smoking decreases the risk of appearance of TPOAb and TgAb and autoimmune hypothyroidism of approximately 40%; this protective effect disappears few years after cessation. It has been suggested that while activating nicotine receptors on immune cells, the autoimmune profile moves away from Th1 and Th17 responses (42, 43).

The proportion of smoking people was more elevated in patients with severe ophthalmopathy (64.2% in GO and 47.9% in GD versus 30% in control subjects) (32, 44–47). Smoking is related to an increased risk of Graves’ hyperthyroidism (twofold), GO (three to four times), or relapse of GD or GO (48, 49), and it is linked to a more severe GO. Many different hypotheses about how smoking may enhance the risk of GD or GO have been reported (50–58).

### Viruses

Viruses activate the adaptive and innate immunity and might cause HT. Human T-cell lymphotropic virus-1, herpes simplex virus, rubella, mumps virus, Epstein–Barr virus, enterovirus in HT, retroviruses [human T-cell lymphotropic virus-1, human foamy virus or human immunodeficiency virus (HIV), and Simian virus 40] in GD have been shown (59). Acquired immunodeficiency syndrome and HIV have been associated with various thyroid diseases (60). Hepatitis C virus (HCV) and human parvovirus B19 (EVB19) are candidate viruses for AITD (59, 61).

#### Parvovirus B19

EVB19 DNA was detected in about 12% of HT cases versus 3% controls (*P* < 0.03), suggesting that acute EVB19 infection could be associated with the appearance of AT (62, 63).

In slides of paraffin-embedded thyroid tissues from 112 patients undergoing thyroidectomy, EVB19 DNA was revealed in thyrocytes (or in lymphocytes), particularly in papillary TC (PTC), leading to hypothesize that EVB19 is implicated in thyroid carcinogenesis (64). In another study, EVB19 was present in 88% PTC tumors (65).

EVB19 was present in thyrocytes in patients with multinodular goiters, thyroiditis, or GD, by immunohistochemistry, PCR or *in situ* hybridization (66, 67). In addition, in primary thyrocytes, after EVB19 NS1 transfection, positive regulatory domain zinc finger protein 1 upregulation was observed (68).

The relationship between EVB19 and different thyroid disorders needs to be investigated (69).

### Chronic HCV Infection and Thyroid

Viral replication occurs in hepatocytes and also in extrahepatic tissues and peripheral blood mononuclear cells (70, 71). Extrahepatic manifestations (72, 73) [mixed cryoglobulinemia (MC), endocrinological diseases (AITD and type 2 diabetes), Sjogren’s syndrome] are present in about 38–76% patients with chronic hepatitis C (CHC). HCV interferes with functions and self-recognition mechanisms (in immune system or in thyrocytes) leading to destruction of thyroid and beginning the autoimmune disease (74).

The association between HCV infection and thyroid autoimmunity has been shown (75–82). Thyroid disorders frequency in interferon (IFN)-free HCV subjects is approximately 10–15% (83–89). Thyroid autoimmune abnormalities frequency was also significantly increased in MC + HCV patients versus controls (AT 35 versus 16%; subclinical hypothyroidism, 11 versus 2%).

In female patients with MC + HCV, or CHC, thyroid disorders were represented by higher risk for AT and hypothyroidism and elevated circulating TPOAb (88, 90).

An increased prevalence of PTC has been shown in CHC, with or without MC, overall when AT was present (91–97), suggesting that AT might be a predisposing condition for TC (95–97). An elevated mortality for TC in HCV patients (98) has been shown, suggesting that it impacts survival of these patients, and giving the advice of a careful thyroid monitoring in HCV patients in presence of thyroid nodules. HCV thyroid infection upregulates CXCL10 expression and secretion of thyroid cells, recruiting other Th1 lymphocytes into the gland, leading to the appearance of AITD in predisposed individuals (99, 100). A careful thyroid function and nodules follow-up in subjects showing risk factors (female gender, a border line high initial TSH, TPOAb+, a small and hypoechoic thyroid) for the appearance of AITD in HCV and MC + HCV should be performed.

### Drugs

Among drugs, IFN- and iodine-containing drugs have been associated with AITD. The “*de novo*” presence of ATA and overt dysfunctions in euthyroidism have been reported upon IFN-α therapy, suggesting that it can exacerbate or induce latent thyroid disorders, inducing AITD (74). The standard dual therapy with pegylated IFN-α/ribavirin has been substituted by a triple therapy with new direct-acting antiviral drugs (NS3/4A serine protease inhibitor) (74) (with/without ribavirin), thereby ameliorating the patient compliance and decreasing the risk for thyroid autoimmunity (74).

Drugs containing iodide may induce hypothyroidism in euthyroid HT patients, 131-I or surgically treated GD, or following hemithyroidectomy for nodules. Medications can also induce hyperthyroidism in patients with endemic iodine-deficient goiter, autonomous nodules, or non-toxic nodular goiter, or in patients recently treated with antithyroid drugs for GD. Rarely, hypothyroidism or hyperthyroidism occur in patients with normal thyroid function in therapy with iodide. The etiology of hypothyroidism and goiter induced by iodide is not clear in patients with cystic fibrosis. Iodine-induced hypothyroidism might appear in patients treated with drugs altering thyroid function (lithium, phenazone, sulfisoxazole, etc.) (101). Amiodarone (for treatment of cardiac arrhythmias) has serious adverse effects on the thyroid function. Amiodarone has similar structure to thyroid hormones, for example, the iodine moieties on the inner benzene ring, and it might cause thyroid dysfunction. Thyroid dysfunctions induced by amiodarone are “amiodarone-induced thyrotoxicosis” (AIT; usually in iodine-deficient areas) and “amiodarone-induced hypothyroidism” (AIH; commonly in iodine-sufficient areas). The type 1 AIT is commonly present in patients with previous thyroid dysfunctions or goiter, while type 2 AIT in normal thyroid and leads to destructive thyroiditis. Patients treated with amiodarone should be controlled for the presence of thyroid dysfunctions. When monitoring patients, initial tests should include TSH, thyroxine (T4), triiodothyronine (T3), and ATA. The treatment depends on the type of AIT, including thionamides and/or glucocorticoids. AIH responds positively to thyroxine replacement therapy (102–104).

### Microbiota

As intestinal dysbiosis occurs, the epithelial barrier fails to function and there is the appearance of intestinal and systemic disorders. The intestinal tract is determinant in metabolizing nutrients, drugs, and hormones, exogenous and endogenous iodothyronines, and micronutrients implicated in thyroid homeostasis. Different autoimmune disorders have a pathogenetic link with dysbiosis, not yet clarified in AITD. It has been hypothesized that intestinal dysbiosis may cause AT. Hyper- and hypothyroidism, frequently in AITD, are associated with bacterial overgrowth in small intestinal or with changes in composition of microbiota (105).

### Vitamin D

Vitamin D is responsible for anti-inflammatory and immunoregulatory effects (106). Different autoimmune diseases show low vitamin D levels, which are also associated with ATA, altered thyroid function, high thyroid volume, elevated TSH levels, and adverse pregnancy outcome in AITD women. Vitamin D binds to its receptor, harbored on many human immune cells, and modulates immune cells activity, provoking innate and adaptive immune responses. Vitamin D receptor gene polymorphisms are associated with AITD, and vitamin D deficiency is linked to AITD through gene polymorphism (in particular *Bsm*I and *Taq*I polymorphism) or environmental factors (lack of dietary uptake and sun exposure) (107). A study evaluated the association between vitamin D levels and AITD through systematic literature review, suggesting that low levels of serum 25(OH)D was related to AITD (108, 109).

### Chemicals

Long-term human studies on the effects of environmental chemicals on thyroid-related outcomes such as growth and development are still lacking. The human exposure scenario with lifelong exposure to a vast mixture of chemicals in low doses and the large physiological variation in thyroid hormone levels between individuals render human studies very difficult. Polychlorinated biphenyls (PCBs) have thyroid-disrupting effects, and it is suggested that also bisphenol A, phthalates, brominated flame retardants, and perfluorinated chemicals show thyroid-disrupting characteristics (110, 111). PCBs, or other persistent organochlorine compounds, disrupt thyroid hormone homeostasis, as shown by animal studies, while dietary exposure to PCBs affects serum thyroid hormones and TSH in human subjects. A study reviewed available epidemiologic studies within this field, reporting discordant results between studies of correlations, and suggesting that there are no clear interstudy dose–response associations. However, current data do not exclude such associations (112). Among the considered studies by a systematic analysis of associations between PCB exposure and thyroid hormones or TSH in pregnant women and newborns, only one showed a significant association between PCBs and total T3 levels, but no association was evidenced assessing thyroid function by serum TSH and free T4 (113, 114).

### Heavy Metals

Cadmium (Cd) is considered a category I carcinogen (on lungs, testicles, and prostate). Cd accumulates in liver, pancreas, kidneys, and also in the thyroid. Cd in blood correlates with concentrations in the thyroid. Cd blood and urine levels are more elevated in women in fertile age, than in men. Mitochondria are the main intracellular targets for Cd. In chronic Cd toxicity, multinodular goiter, thyroglobulin hyposecretion, and parafollicular cell hyperplasia are frequent (115).

Manganese (Mn) is toxic at high levels. Oxidoreductases, transferases, hydrolases, lyases, isomerases, ligases, glutamine synthetase, and Mn superoxide dismutase are Mn-containing enzymes. Mn superoxide dismutase is the main antioxidant enzyme able to neutralize the toxic effects of reactive oxygen species. Environmental or occupational exposure to high levels of Mn causes a neuropathy similar to idiopathic Parkinson’s disease, known as manganism. Dopamine and its metabolites are altered in manganism and Parkinson’s disease, as they are able to inhibit TSH secretion. Dopamine and dopaminergic receptors are involved in neurodevelopment and TSH modulation. This suggests that excessive Mn exposure during gestation is linked to altered neurodevelopmental outcomes, due to a dysregulation of dopaminergic control of TSH on thyroid hormones levels (116).

Thyroid cancer incidence is markedly increased in volcanic areas. In the volcanic area of Mt. Etna in Sicily, a non-anthropogenic pollution with heavy metals has been documented, a consequence of gas, ash, and lava emission. Soil, water, and atmosphere contamination, *via* the food chain, biocontaminate the residents as documented by high levels in the urines and the scalp hair compared to individuals living in adjacent non-volcanic areas. Trace amounts of metals are essential nutrients but, at higher concentrations, can be toxic for living cells. Metals can behave both as endocrine disruptors, perturbing the hormonal system, and as carcinogens, promoting malignant transformation. Similar to other carcinogens, the transforming effect of heavy metals is higher in developing organisms, such as the fetus (contaminated *via* the mother), and individuals in early childhood. The metal concentration in tissues has been rarely measured in the thyroid (117).

## Conclusion

Although the genetic background accounts for approximately 70% of the risk for developing AITD, environmental triggers have an important role in the AITD development in susceptible individuals. Radiation exposure, and increased iodine intake, selenium, and vitamin D deficiency are environmental factors increasing the AITD risk. GO and GD are associated with cigarette smoke (50, 118), while it seems to decrease the risk of hypothyroidism and prevalence of thyroid antibodies (42, 43). Viral infections are a determinant environmental factor in AITD pathogenesis (1, 10). Among the many chemical contaminants, halogenated organochlorines and pesticides variably disrupt thyroid function. PCBs and their metabolites and polybrominated diethyl ethers, bind to thyroid transport proteins, such as transthyretin, displace thyroxine, and disrupt thyroid function. Meanwhile, at the molecular level, PCB congeners may result in inhibition of the natrium/iodide symporter (119). Among drugs, IFN- and iodine-containing drugs have been associated with AITD. Moreover, it seems that intestinal dysbiosis triggers AT (105). To reduce the risk to populations but also in each patient, it is necessary to comprehend the association between environmental agents and thyroid dysfunction. These factors are considerable for those subjects who are at increased risk of AITD according to their family history (120).

## Author Contributions

FSM, FP, AA, and BS gave substantial contribution in the conception and design of the work, and in writing the paper; gave the final approval of the version to be published; agreed to be accountable for all aspects of the work in ensuring that questions related to the accuracy or integrity of any part of the work are appropriately investigated and resolved. AA and BS revised it critically for important intellectual content.

## Conflict of Interest Statement

The authors declare that the research was conducted in the absence of any commercial or financial relationships that could be construed as a potential conflict of interest.

## Abbreviations

AITD, autoimmune thyroid diseases; GD, Graves’ disease; HT, Hashimoto’s thyroiditis; ATA, antithyroid antibodies; TPOAb, thyroid peroxidase antibodies; GO, Graves’ ophthalmopathy; TgAb, thyroglobulin antibodies; AT, autoimmune thyroiditis; TSH, thyroid-stimulating hormone; HIV, human immunodeficiency virus; HCV, hepatitis C virus; EVB19, erythrovirus B19; TC, thyroid cancer; PTC, papillary thyroid cancer; CHC, chronic hepatitis C; MC, mixed cryoglobulinemia; IFN, interferon; AIH, amiodarone-induced hypothyroidism; AIT, amiodarone-induced thyrotoxicosis; PCB, polychlorinated biphenyl; T3, triiodothyronine; T4, thyroxine; Cd, cadmium; Mn, manganese.
